# Nanostructured Fe,Co-Codoped MoO_3_ Thin Films

**DOI:** 10.3390/mi10020138

**Published:** 2019-02-20

**Authors:** Olfa Kamoun, Amel Mami, Mohamed Aymen Amara, Ruxandra Vidu, Mosbah Amlouk

**Affiliations:** 1 Department of Physics, University of Tunis El Manar, 2092 Tunis, Tunisia; mohamedaymen08@gmail.com (M.A.A.); mosbah.amlouk@gmail.com (M.A.); 2 UR Photothermy, Photothermal Laboratory, Preparatory Institute for Engineering Studies of Nabeul (IPEIN), 8000 Merazka, Nabeul, Tunisia; mamiamel@gmail.com; 3 Department of Electrical Engineering and Computer Science, University of California, Davis, CA 95616, USA; rvidu@ucdavis.edu; 4 Faculty of Materials Sciences, University Politehnica of Bucharest, UPB-ECOMET, 011061 Bucharest, Romania

**Keywords:** spray pyrolysis, thin films, MoO_3_, X-ray diffraction, morphology, optical properties, transmission electron microscopy (TEM), thermal, photocatalysis

## Abstract

Molybdenum oxide (MoO_3_) and Fe,Co-codoped MoO_3_ thin films obtained by spray pyrolysis have been in-depth investigated to understand the effect of Co and Fe codoping on MoO_3_ thin films. The effect of Fe and Co on the structural, morphological and optical properties of MoO_3_ thin films have been studied using X-ray diffraction (XRD), scanning electron microscopy (SEM), transmission electron microscopy (TEM) and energy-dispersive X-ray analysis (EDAX), optical and photoluminescence (PL) spectroscopy, and electropyroelectric methods. The XRD patterns demonstrated the formation of orthorhombic α-MoO_3_ by spray pyrolysis. SEM characterization has shown an increase in roughness of MoO_3_ thin films by Fe and Co doping. Optical reflectance and transmittance measurements have shown an increase in optical band gap with the increase in Fe and Co contents. Thermal conductivity and thermal diffusivity of Fe,Co-doped MoO_3_ were 24.10–25.86 Wm^−1^K^−1^ and 3.80 × 10^−6^–5.15 × 10^−6^ m^2^s^−1^, respectively. MoO_3_ thin films have shown PL emission. Doping MoO_3_ with Fe and Co increases emission in the visible range due to an increase number of chemisorbed oxygen atoms. The photodegradation of an aqueous solution of methylene blue (MB) depended on the content of the codoping elements (Fe,Co). The results showed that a degradation efficiency of 90% was observed after 60 min for MoO_3_: Fe 2%-Co 1%, while the degradation efficiency was about 35% for the undoped MoO_3_ thin film.

## 1. Introduction

Recently, nanosize molybdenum oxide has showed interesting applications. The investigation of α-MoO_3_ nanorods obtained by hydrothermal process has shown that nanorods of about 10 μm in length and 200–300 nm in diameter can be obtained. These nanorods exhibited a significant response against triethylamine vapor with a concentration of 0.1 ppm at 300 °C [1]. Investigation of MoO_3_ catalyst in the conversion of furfuryl alcohol (FA) and its selectivity towards dimers (C_9_-C_10_) and trimers (C_14_-C_15_) has shown that the FA conversion increased with the reaction time, but the selectivity decreases at longer reaction times [2]. Investigations of MoO_3−x_ nanodots for medical applications have shown that the nanosized molybdenum oxide may act as a potential therapeutic material for the treatment of amyloid induced neurotoxicity [3].

The α-MoO_3_ film obtained by spray pyrolytic deposition was recently investigated for its use in dye-sensitized solar cell (DSSC) [4]. Although MoO_3_ films have been extensively investigated for their catalytic properties for the chemical industry and environmental remediation, only a few papers have dealt with the investigation of molybdenum oxide as counter electrode in DSSC. In fact, more attention has been paid to the study of the photocatalytic activity of the MoO_3_ in a powder form [5,6,7]. In contrast, a few researches have been published describing photocatalytic performance of the molybdenum oxides thin films [8,9]. In addition, MoO_3_ nanoparticles have been synthesized using a hydrothermal method and it was shown that the methylene blue removal is promoted by adsorption instead of photocatalytic mechanisms [6].

Over the past decade, photocatalysis technologies is the most promising for environmental purification and conversion of solar energy and ultra-violet (UV) [10,11,12,13]. Multifunctional properties such as a combination of optical, semiconducting and catalytic of metal oxides (ZnO [14] and MoO_3_) thin films have been recently investigated. Photocatalytic properties of thin films have not been much studied until recently. M. Ponce-Mosso et al. [9] prepared amorphous MoO_3_ thin films by radio frequency (RF) reactive magnetron sputtering, using a Mo target. The optimum photocatalytic activity was found for MoO_3_ films deposited at different sputtering power and working pressure [9]. Molybdenum oxides are exciting materials with various applications such as optoelectronics, catalysis, sensors, superconductors, biosystems, and electrochromic systems. These oxides are obtained in several stoichiometries, among which is the MoO_3_ stoichiometric compound with relatively wide bandgap energy (*E_g_* = 3 eV [15,16,17]. MoO_3_ may crystalize in various crystal structures such as orthorhombic, monoclinic and hexagonal, depending on how they share the MoO_6_ octahedra, i.e., sides or corners). There are two basic polytypes of MoO_3_: one is the orthorhombic MoO_3_ (type R), which is a phase thermodynamically stable, and another one is a metastable monoclinic MoO_3_ (α-type) with a ReO_3_ type structure. The orthorhombic MoO_3_ phase [18] is well known as a compound layered in two-dimensional planes.

Recently, molybdenum oxides have synthesized in various nano-forms such as nanorods, nanobelts, nanopores, and ultra-thin films. MoO_3_ ultrathin films are used in smart windows and electrochemical systems. A complete overview of the structure of undoped and doped thin films based on MoO_3_ are reported elsewhere [18,19,20]. In our group, MoO_3_ thin films doped with Co and Ni [21] and Eu [20] obtained by spray pyrolysis have been throughout investigated. In this work, in order to enhance photocatalytic activity of pure MoO_3_ thin films, we codoped MoO_3_ thin films with iron and cobalt. In our knowledge, there are no studies reported in the literature on MoO_3_ thin films codoped with iron and cobalt. This work presents photocatalytic behavior and physical investigations of MoO_3_ thin films codoped with iron and cobalt obtained by spray pyrolysis. Specific emphases are put on the thermal as well as the photosensitivity [19,22,23,24,25,26,27,28,29,30] of such codoped films against Methyl blue dye (MB). Photocatalysis application seems to be sensitive to an appropriate codoping ratio. Our goal to use thin films is making MoO_3_ with a rough surface that consequently increases the specific surface and can be used for photocatalysis micromachines.

## 2. Experimental Method

### 2.1. Fe-Co Codoped MoO_3_ Thin Films Deposition

Thin films deposition was performed by spray pyrolysis at 460 °C on a glass substrate using the deposition conditions detailed by Boukhachem et al. [31]. The spraying solution consisted of 0.01 M aqueous solution of ammonium molybdate tetrahydrate [(NH_4_)6Mo_7_O_24_4H_2_O], and the source of iron and cobalt was iron (II) sulfate hexahydrate (FeSO_4_, 6H_2_O) and cobalt (II) chloride hexahydrate (CoCl_2_, 6H_2_O), respectively. The molar ratios (Fe/Mo) and (Co/Mo) were 0, 1 and 2%. The gas carrier was blown with nitrogen through a nozzle 0.5 mm in diameter at a pressure of 0.35 bar. The gas carrier was nitrogen, which was blown through a 0.5 mm-diameter nozzle at a pressure of 0.35 bar. The flow rate of the precursor mixture was 6.67 × 10^−5^ l/s during deposition. Following the deposition, the films were allowed to cool.

### 2.2. Techniques Used for the Fe-Co Codoped MoO_3_ Thin Film Characterization

The crystallographic structure of the films was studied using a Philips PW 1729 X-ray diffractometer with Cu-Ka monochromatic radiation (*λ* = 0.15405 nm). A Perkin-Elmer spectrophotometer was used to study optical reflectance *R(λ)* and transmittance *T(λ)* within the wavelength range from 200 to 2000 nm. Scanning electron microscopy (SEM) with EDAX was used to investigate the morphology of the thin films. To determine thermal parameters of such thin films, we have used ElectroPyroElectric (EPE) technique. The measurements were performed using an excitation source in the form of modulated electrical current to generate a photothermal signal. Thermal parameters of the pyroelectric cell were reported in [31].

The photocatalytic decomposition of methylene blue (MB) was measured using two UV lamps in parallel with a total power of 16 W. The thin film sample with an area of 1 cm × 3 cm was placed in 25 mL volume of aqueous solution containing 3 mg/L MB. To establish an adsorption–desorption equilibrium, the solution was magnetically stirred in the dark for at least 30 min prior to the experiment. UV-vis spectrometer was used to quantitatively evaluate the decomposition of MB after UV illumination.

## 3. Structural Investigation

### 3.1. X-ray Diffraction Analyses

**Figure 1:**
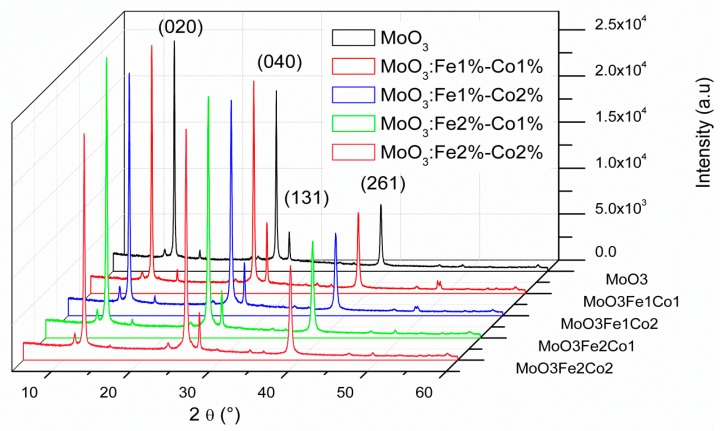
X-ray diffraction patterns of (Co,Fe)-doped MoO_3_ thin films.

Figure 1 shows the XRD spectra of (Fe, Co) doped MoO_3_ thin films for different doping concentrations. The peaks corresponding to the (020), (040), (131), and (261) planes agree with the orthorhombic α-MoO_3_ structure (JCPDS card#: 76-1003), having *a* = 3.96 Å, *b* = 13.86 Å, *c* = 3.7 Å, and preferential orientations in the (020) and (040) directions [32,33]. The intensity of the main peak (020) increases with Fe and Co contents up to (Fe 2%, Co 1%) which indicates that the crystallinity of the MoO_3_ thin films increases by doping. The improvement of the crystallinity with the Fe-Co codoping can be explained as follows: the Fe-Co codopants are placed in the substitutional sites which have the effect on improving the structure with an optimum for Fe 1%-Co 2%, beyond codoping Fe 1%-Co 2% it can be said that Fe-Co codopant elements have the effect of reducing the crystallinity.

Additional information on the Fe-Co doping effect on the MoO_3_ thin films structure was obtained by further analysis of the XRD scans. The interplanar distance *d_hk_*_l_ of MoO_3_: Fe-Co thin films was calculated using the Bragg equation as follows:

(1) $$2d_{hkl}sin\theta =n\lambda$$

where *n* is a positive integer, λ is the wavelength of the incident wave.

The values calculated for the interplanar distance *d_hkl_* for MoO_3_: Fe-Co thin films are presented in Table 1. Analyzing the results obtained for MoO_3_ thin films with different Fe-Co content, we observe that Fe-Co doping does not affect the *d_hkl_* values. Therefore, we can assume that Fe and Co ion are substitutional dopants and do not occupy interstitial sites.

Further, we calculated the lattice parameters *a*, *b* and *c* from the *d_hkl_* values presented in Table 1 using the following relation [34]:

(2) $$\frac{1}{d_{hkl}^{2}}=\frac{h^{2}}{a^{2}}+\frac{k^{2}}{b^{2}}+\frac{l^{2}}{c^{2}}$$

and the texture coefficient *TC*(*hkl*), which gives the preferred orientation of the film, with the following relation [20,35,36]:

(3) $$TC\left(hkl\right)=\frac{I\left(hkl\right)/I_{0}\left(hkl\right)}{N^{-1}\sum_{n}I\left(hkl\right)/I_{0}\left(hkl\right)}$$

where *I*(*hkl*) and *I*_0_(*hkl*) are the measured and the standard intensity of the plane (*hkl*), respectively, and *N* is the reflection number.

Table 2 shows the *TC*(*hkl*) values calculated for the MoO_3_: Fe-Co thin films, where the highest values are for TC(020) and TC(040). These results indicate that the thin films are formed of crystallites parallel to the (0k0) planes.

The best crystallinity is obtained for MoO_3_: Fe 2%-Co 1% with preferentially orientation along (020) direction. The effect of Fe-Co dopants on the MoO_3_ crystal lattice can be further analyzed by studying the crystallite size (*D*), the stress *ξ* and dislocation density *δ_dis_* [35,37,38,39,40] with doping, according to the following equations:

(4) $$D=\frac{k\lambda }{\beta_{1/2}cos\theta }$$

(5) $$\xi =\frac{\beta cos\theta }{4}$$

(6) $$\delta_{dis}=\frac{1}{D^{2}}$$

where *k* is the Scherrer constant (*k* = 0.90), *β*_1/2_ is the half width of the peak (corrected value). Table 3 presents the values obtained *D*, *ξ* and *δ_dis_* along (020) calculated using Equations (4)–(6).

These results show that both *ξ* and *δ_dis_* values increase with Fe-Co codoping, while the crystallite size *D* shows a decrease. The codoping does not affect the lattice parameters but influences the *D*, *ξ* and *δ_dis_* values. The lowest *D* value at 65.1 nm is obtained for MoO_3_: Fe 2% Co 1%, then specific surface has increased. This result recommends the material for photocatalytic application. The highest values of the stress *ξ* at 55 × 10^−4^ and dislocation density *δ_dis_* at 24 × 10^13^ lines/m^2^ are obtained for the same material, i.e., MoO_3_: Fe 2% Co 1%; the high values for *ξ* and *δ_dis_* may be responsible for the reduced crystallite size. We can explain the highest values of *ξ* and *δ_dis_* and the lowest value of *D* obtained for MoO_3_: Fe 2% Co 1%, by the fact that the substitution of molybdenum cation by iron and cobalt cations is saturated at this codoping ratio and any additional Fe and Co cations take interstitial sites.

### 3.2. SEM-EDAX Characterization

**Figure 2:**
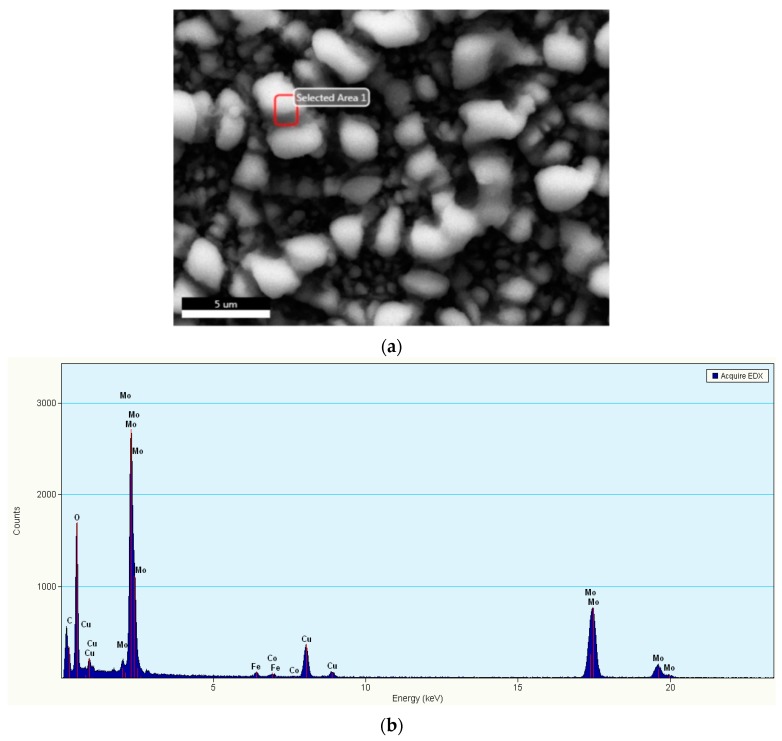
(**a**) Select area for energy-dispersive X-ray analysis (EDAX), (**b**) EDAX spectra of the MoO_3_ thin film codoped Fe 2% Co 1% showing the presence of the characteristic elements.

The elemental analysis of the doped MoO_3_ thin film was performed using EDAX spectra under SEM. The presence of peaks corresponding to Mo, Fe, Co and oxygen confirms the formation of MoO_3_ codoped with both Fe and Co elements, Figure 2a,b. Figure 2b shows the EDX spectra of the MoO_3_ obtained for the selected SEM area in Figure 2a. The characteristic energy lines for oxygen and molybdenum are located at the energies presented in Table 4. The peaks corresponding to O Kα and Mo Lα1 lines are the most intense. The composition of the film was calculated by computer software taking into account the energy lines of O K and Mo L lines for MoO_3_, which corresponds to the ionized electronic shells [37].

**Figure 3:**
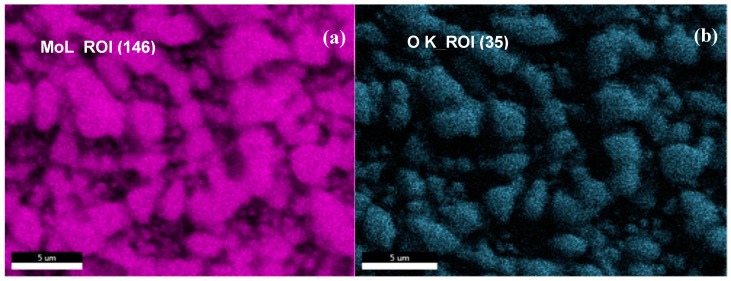

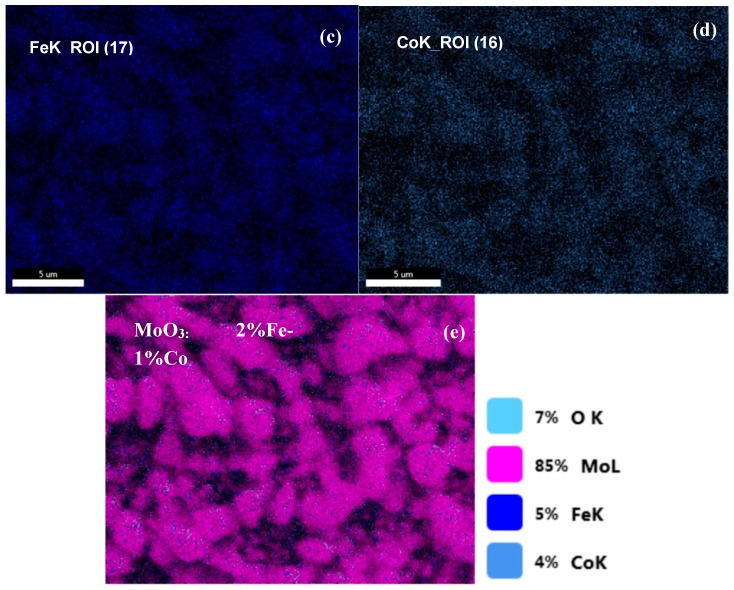
Scanning electron microscopy (SEM) elemental mapping of the MoO_3_ surface; (**a**) pink color shows molybdenum atoms; (**b**) light blue color shows oxygen atoms; (**c**) navy blue color represents Fe present on the surface, (**d**) grey color shows the Co atoms and (**e**) MoO_3_ surface mapping showing the distribution of all the elements on the surface.

The SEM elemental mapping of MoO_3_: Fe 2%-Co 1% film is shown in Figure 3 for Mo, O, Co, Fe and a map of all of them superimposed elements. The film morphology is characterized by nanoparticles uniform distributed over the surface. The energy dispersive spectroscopy (EDS) and the elemental mapping of the doped MoO_3_ film demonstrate the homogeneous distribution of Mo and O elements (Figure 3a,b). Furthermore, the image presented in Figure 3e as a superimposition of the elemental mapping of all elements shows a uniform dispersion of MoO_3_ nanoparticles.

### 3.3. SEM and TEM Observations

**Figure 4:**
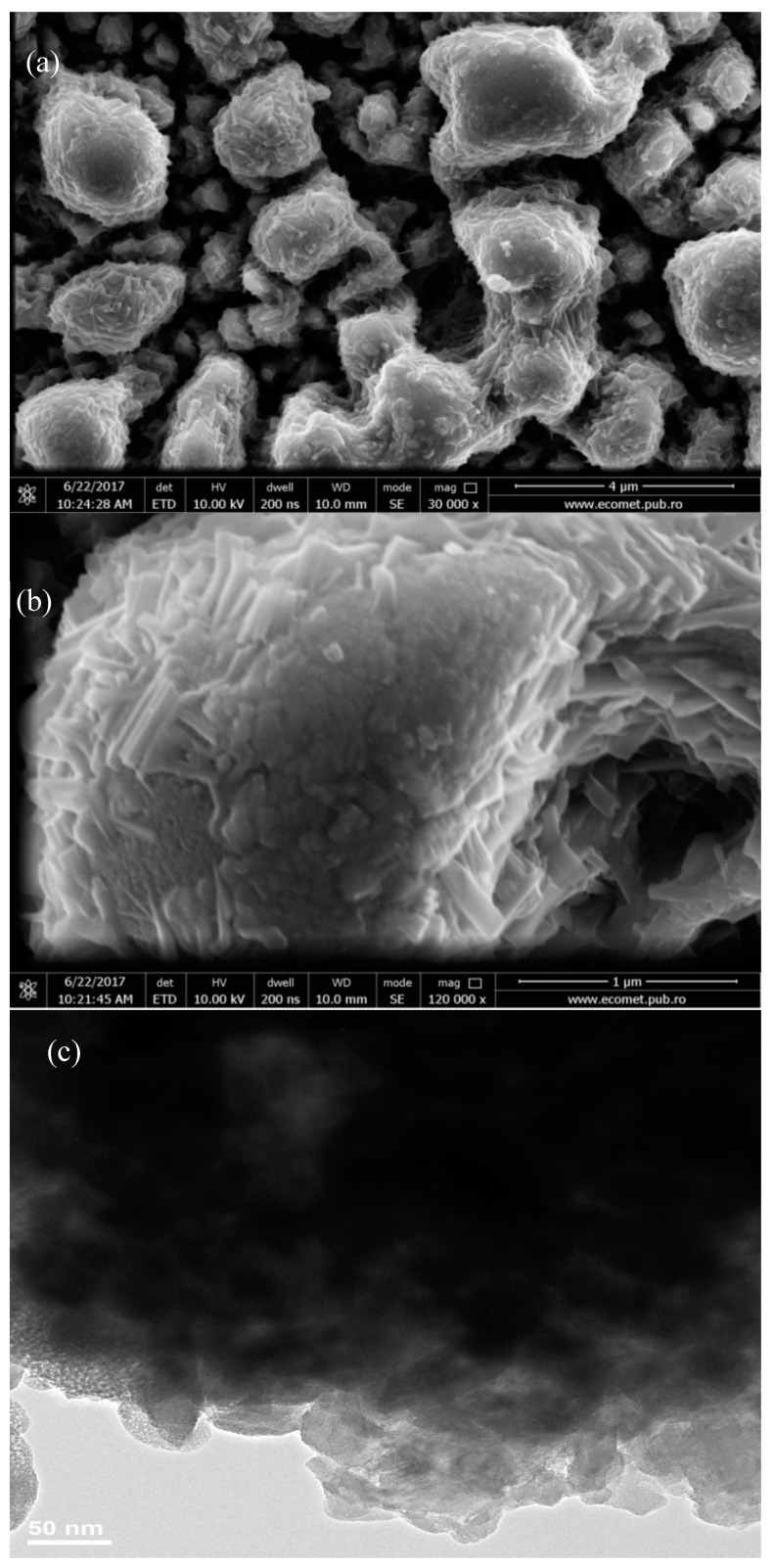
SEM images at 30,000× (**a**) and 120,000× (**b**) magnifications and TEM images (**c**) of the codoped MoO_3_: Fe 2%-Co 1% thin films.

**Figure 5:**
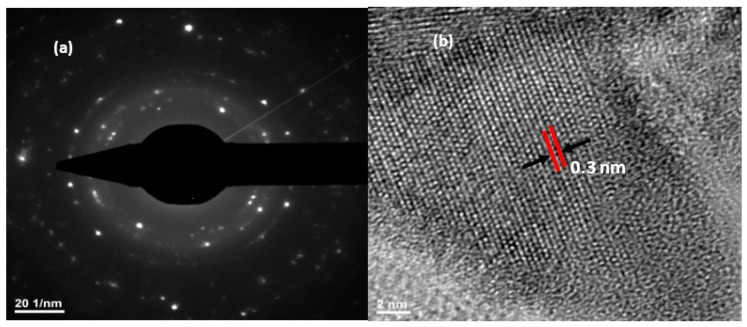
SAED pattern (**a**) and HRTEM image (**b**) of MoO_3_: Fe 2%-Co 1%.

The SEM, TEM, high-resolution transmission electron microscopy (HRTEM), and selected area (electron) diffraction (SAED) images of MoO_3_: Fe 2%-Co 1% are shown in Figure 4 and Figure 5. Figure 4 shows a typical SEM images of α-MoO_3_ film obtained by spray pyrolysis. Similar morphology of α-MoO_3_ nanoplates has been observed for molybdenum oxide thin films obtained by this method [20,21]. TEM image presented in Figure 4c shows that most of α-MoO_3_ plates present a four-sided plate-like shape with a length of 20–50 nm. Also, the plates seem to be stacked one over the other, the overlap being clearly seen in the SEM images.

The SAED pattern presented in Figure 5a has been indexed to α-MoO_3_. Figure 5b shows the HRTEM image of the α-MoO_3_, which was taken at the edge of the plate. The HRTEM image clearly indicates that the α-MoO_3_ nanoplate is locally a single-crystal. The distance between the two-dimensional lattice stripes is about 0.3 nm, which is similar to that reported by Li et al. [39]. The diffraction pattern obtained from HRTEM and SAED images are similar, but the distance between the α-MoO_3_ stripes differs from the 0.3 nm. The plate-like morphology of the α-MoO_3_ phase is characterized by a large side-to-thickness ratios with the large surface parallel to the substrate. This morphology could cause certain defects in the structure, which could explain the distance difference between stripes when the HRTEM and SAED measurements are compared. Taking into consideration the plate morphology together with the XRD observation on preferred growth of (0k0) planes, we can conclude that the plate-like MoO_3_ crystal have the shortest side direction along the b-axis, which means that the direction of the nanoplate thickness is along the b-axis.

## 4. Optical Investigations

**Figure 6:**
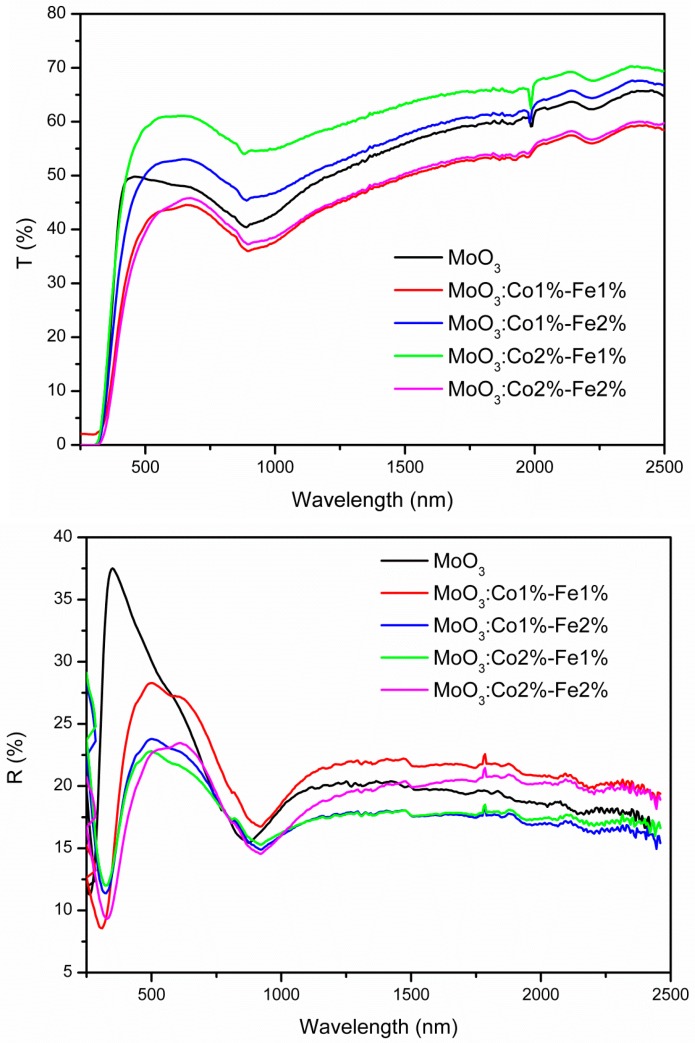
Transmission (T%) and reflection (R%) spectra of MoO_3_: Fe-Co thin films.

Optical investigation of the thin films was performed by measuring the transmission and reflectance. The optical transmission spectra are presented in Figure 6. In the visible range, the average transmittance of the thin films was between 40 and 75%, while the reflectance was between 7 and 37%. We observed that *T(λ)* and *R(λ)* spectra varies with Fe-Co content and the highest transmission in the visible range is obtained for MoO_3_: Fe 2%-Co 1%. Since the transitions of electrons from the valence to the conduction band corresponds to the absorption edge, the effect of doping concentration on the optical band gap of the films can be calculated. The absorption coefficient can be expressed according to the following equation [21,41]:

(7) $$\alpha =\frac{1}{d}Ln\frac{{\left(1-R\right)}^{2}}{T}$$

**Figure 7:**
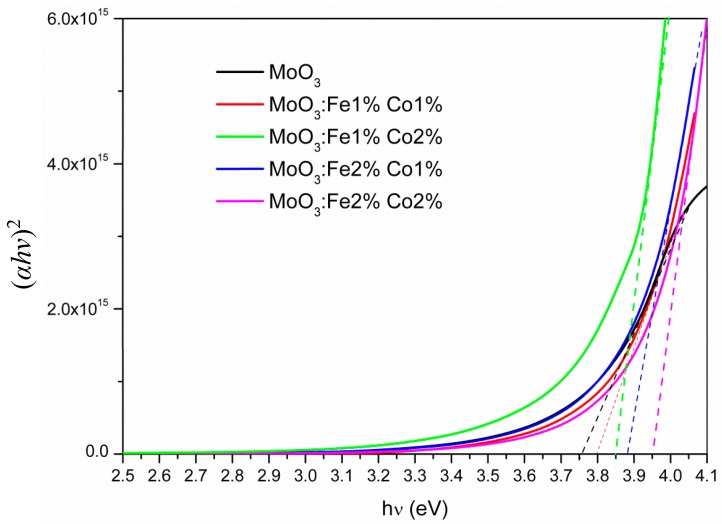
The (*αhν*)^2^ plot vs. *hν* for MoO_3_: Fe-Co thin films.

For a direct band gap semiconductor, the following relationship exists between band gap and absorption coefficient [42]:

(8) $$\alpha h\nu =B{\left(h\nu -E_{g}\right)}^{p}$$

where *B* is a constant, *E_g_* is the optical band gap, *hν* is the incident photon energy, and *p* is a number which is equal to ½ for direct transition. From the (*αhν*)^2^ versus *hν* plots, the optical band gap *E_g_* can be determined where the tangent to the curve intersect the *x*-axis (Figure 7). The calculated values of *E_g_* of undoped and Fe-Co doped MoO_3_ are summarized in Table 5. We observed that the *E_g_* value increase by Fe-Co doping and the highest band gap was obtained for MoO_3_: Fe 2%-Co 2%. Additionally, at energies lower than the optical gap, the optical absorption due to crystalline defects appears. Unlike crystalline structures where the adsorption edge is dictated by the difference in the valence and conduction levels, a particular optical absorption edge profile was observed by Mott et al. [43] in the ion-doped binary semiconductor compounds. The high optical transmission observed for MoO_3_: Fe 2%-Co 1% means that the light penetrates better in the thin film, which can increase the total photogenerated electron-hole pairs and subsequently improves the catalyst activity.

For MoO_3_ thin film, the absorption coefficient profile increases exponentially with the photon energy near the energy gap [20]. Accordingly, the band gap becomes narrow which is due to the “blurring” effect in valence and conduction bands, and the appearance of the so-called Urbach tailing [20]. Because of these interactions, the absorption coefficient is expressed by the empirical Urbach law [21,42] as follows:

(9) $$\alpha =\alpha_{0}exp\left(\frac{h\nu }{E_{U}}\right)$$

where *α*_0_ is a constant and *E_U_* is the Urbach energy. The *E_U_* values presented in Table 5 show a decrease with the amount of the Fe-Co doping. This can be explained by the improvement observed in the crystallinity by Fe-Co doping as it was shown in the XRD analysis described above. The decrease of *E_U_* by increasing Fe-Co content may be explained by a high density of states in the forbidden band, which may increase the band gap.

## 5. Electropyroelectric (EPE) Investigation

A literature search on the electropyroelectric behavior of the metal-oxide binary systems has provided a little information despite the use of MoO_3_ in dye-sensitized solar cell. Because of certain possible uses of MoO_3_ in photosensitivity applications such as thermoelectric and photocatalysis, we have investigated the thermal behavior of these thin films. The following thermal parameters have been investigated: specific heat, *C*, thermal conductivity *K*, thermal diffusivity *D* and effusivity, *e*. The relationship between these parameters are as follows:

(10) $$C=\frac{k}{D}$$

(11) $$e={\left(Ck\right)}^{1/2}$$

**Figure 8:**
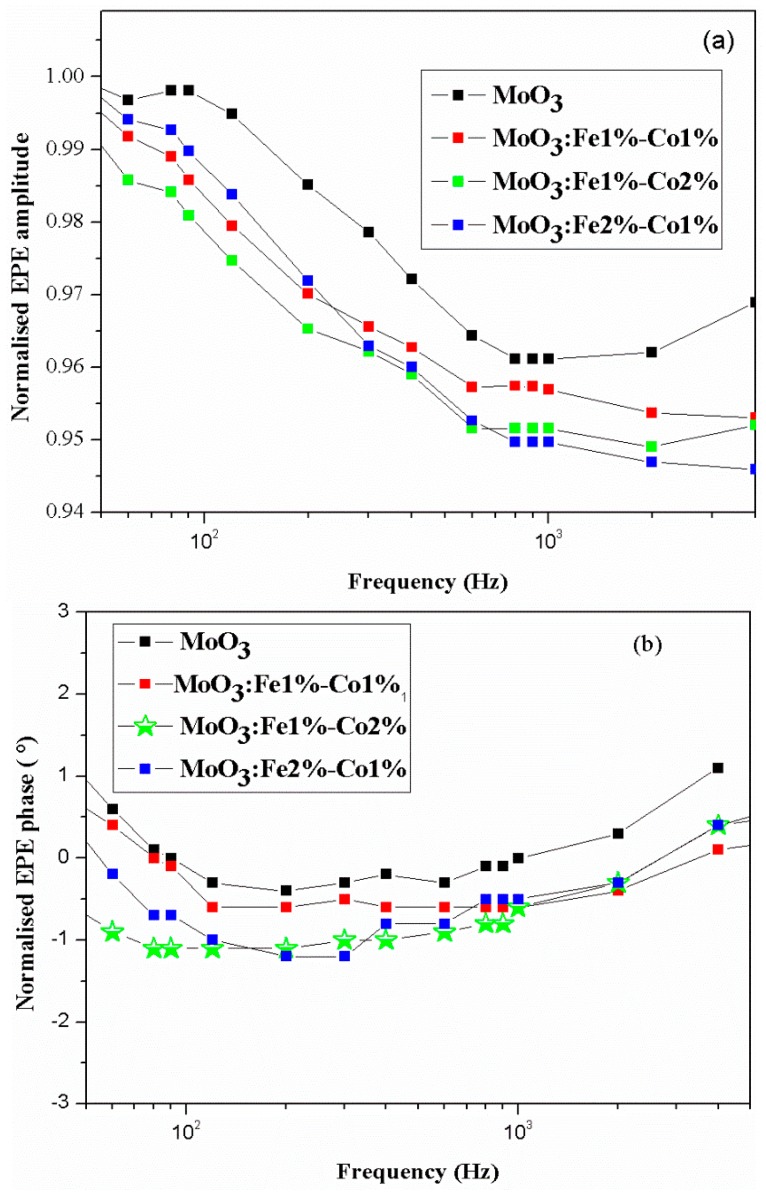
Experimental normalized amplitude (**a**) and phase (**b**) of the electropyroelectric signal for MoO_3_ for different Fe and Co doping amount. EPE: ElectroPyroElectric.

The thermal conductivity and diffusivity of the thin films were measured using the ElectroPyroElectric (EPE) technique in the front detection configuration. The EPE cell used in our experiments consists of five adjacent layers as shown in reference [34]. The theoretical model of the EPE normalized voltage is a complex function that depends on the detector and frequency [44]. Figure 8 show the experimental results on normalized EPE amplitude and phase, obtained for the Co and Fe doped MoO_3_ thin films.

**Figure 9:**
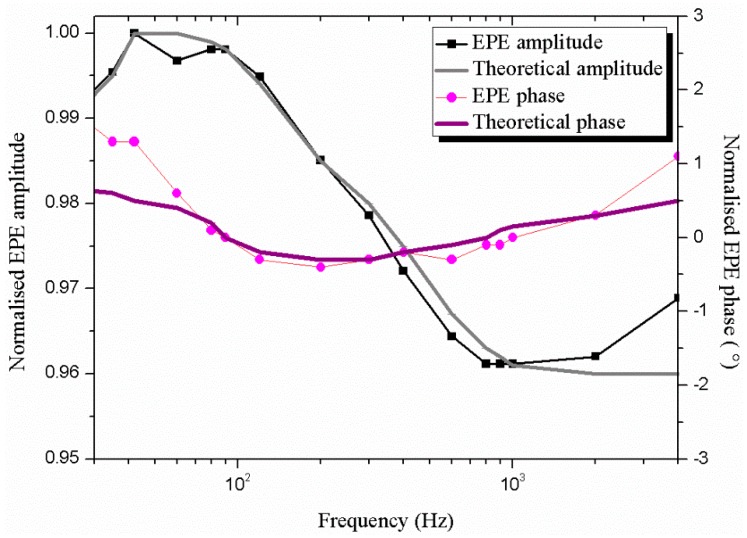
Experimental (dots) and theoretical (line) normalized amplitude and phase of the electropyroelectric signal according to the frequency modulation for MoO_3_ sample.

Figure 9 depicts the best fit of the experimental data for the MoO_3_ film as a function of the modulation frequency. Then, the thermal effusivity *e* and the volume heat capacity *C* can be obtained from Equations (10) and (11). The data obtained from fitting the experimental data with the theoretical values are listed in Table 6. The errors have had a rectangular distribution and were calculated statistically. The thermal conductivity for the Fe-Co codoped MoO_3_ thin films shows an increased trend with the increase in Fe-Co codoping concentration (Table 6). The thermal diffusivity of MoO_3_ is slightly lower than for Fe-Co codoped MoO_3_. Consequently, the rate of the heat transfer of MoO_3_ doped with 2% Fe and 1% Co is high. Thus, despite showing promise, MoO_3_ film cannot surpass the thermal performance of the doped films. In addition, we observed that MoO_3_ doped with 2% Fe and 1% Co was the best material for thermal applications, thanks to its high thermal behavior. Unfortunately, there is not much information on the thermal properties of molybdenum trioxide thin films. Thermal conductivity of molybdenum trioxide thin film sample that ranges from 24.1 to 25.86 Wm^−1^K^−1^ is in good agreement with other high band gap, thermally conductive semiconductors, such as Si_3_N_4_ (~27 W/mK) and BN (~20 W/mK), which are unstable in an oxidizing atmosphere and expensive to produce [45,46]. In fact, the molybdenum oxides exhibited both good Seebeck coefficient of 845 μV/K and electrical conductivity of 0.9 × 10^5^ S/m at 80 °C [18]. Furthermore, the MoO_3_ pure has an impressive thermal diffusivity of 3.8 × 10^−6^ m^2^s with good thermal conductivity of 24.1 W/mK. Because of their thermal and electrical properties, the molybdenum oxides could be used as high-temperature thermoelectric material.

## 6. Photoluminescence

**Figure 10:**
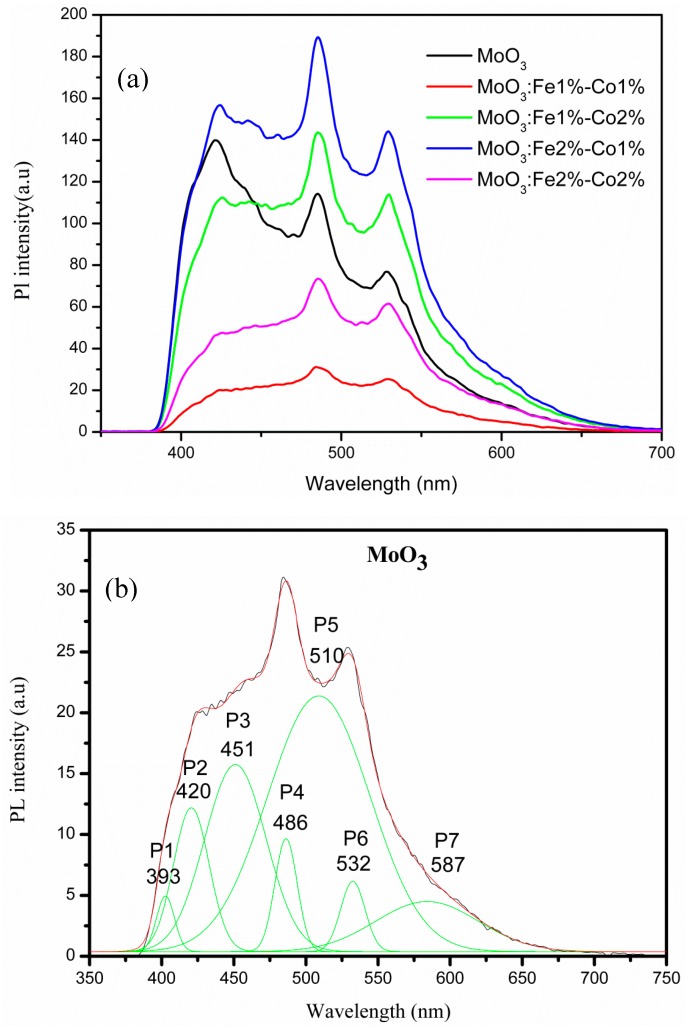
Photoluminescence of MoO_3_: Fe-Co (**a**) and (**b**) Gaussien convolution of PL spectra undoped MoO_3_.

MoO_3_ thin films exhibit PL emission. Figure 10 presents the photoluminescence of doped MoO_3_ along with the multi-peak Gaussian fitting of the undoped MoO_3_ PL spectrum. The peaks labeled Pi, I = 1, 2, 3, …, 7 are located at the following wavelengths: 393, 420, 451, 486, 510, 532, and 587 nm, respectively. Molybdenum oxide exhibits luminescence peaks due to the radiative decay of self-trapped excitons. The traps are associated with certain intrinsic defects such as oxygen vacancies, or even more complex clusters of oxygen vacancies, which affects the molybdenum ion valence related to the charge transfer from O vacancies to Mo [47,48]. The peaks Pi, I = 1, 2, …, 7 presented in Figure 10b on the Gaussian convoluted PL spectra of MoO_3_ corresponding to the free excitons recombination are in agreement with the published literature data [48,49]. The transitions positioned at 451, 486, 510, and 532 nm could be associated with the Mo^6+^ d–d band transition [49,50,51].

The broad features of the PL peaks may be due to the band bending effect at the film surface. Since MoO_3_ is an oxygen sensor with high affinity towards oxygen at room temperature, chemisorptions of oxygen occur when MoO_3_ films is exposed to air. The oxygen adsorption will capture the electrons from the surface, resulting in band bending [52]. When excited, the photogenerated electrons near the surface move across the depletion region in the opposite directions, which reduces their chances of recombination [52]. Simultaneously, the holes move to the surface where they interact with the adsorbed oxygen ions. The physisorbed oxygen is less stable than the adsorbed oxygen and therefore is likely to be desorbed from the surface, thus reducing the band gap and emitting in the visible range. which results in visible emission. Doping MoO_3_ with Fe and Co increases emission in the visible range due to an increase number of chemisorbed oxygen atoms. The PL spectrum of the film presented in Figure 10 shows an interesting aspect in that the intensity of the peak in the visible range is stronger than the UV near-band-edge peak. When the film is Fe-Co codoped, the intensity of the emission peak is stronger compared to the undoped MoO_3_ film. The largest PL peaks where observed for MoO_3_: Fe 2%-Co 1%this can be due to an increase of oxygen vacancy by codoping. This observation leads to the conclusion that the UV and visible luminescence centers are not related.

## 7. Photocatalytic Performance

**Figure 11:**
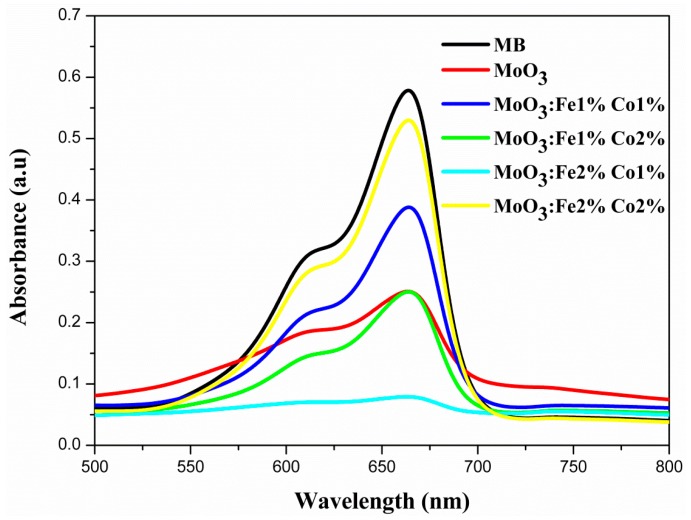
Absorption spectra of methylene blue (MB) solution under UV light after 1 h.

**Figure 12:**
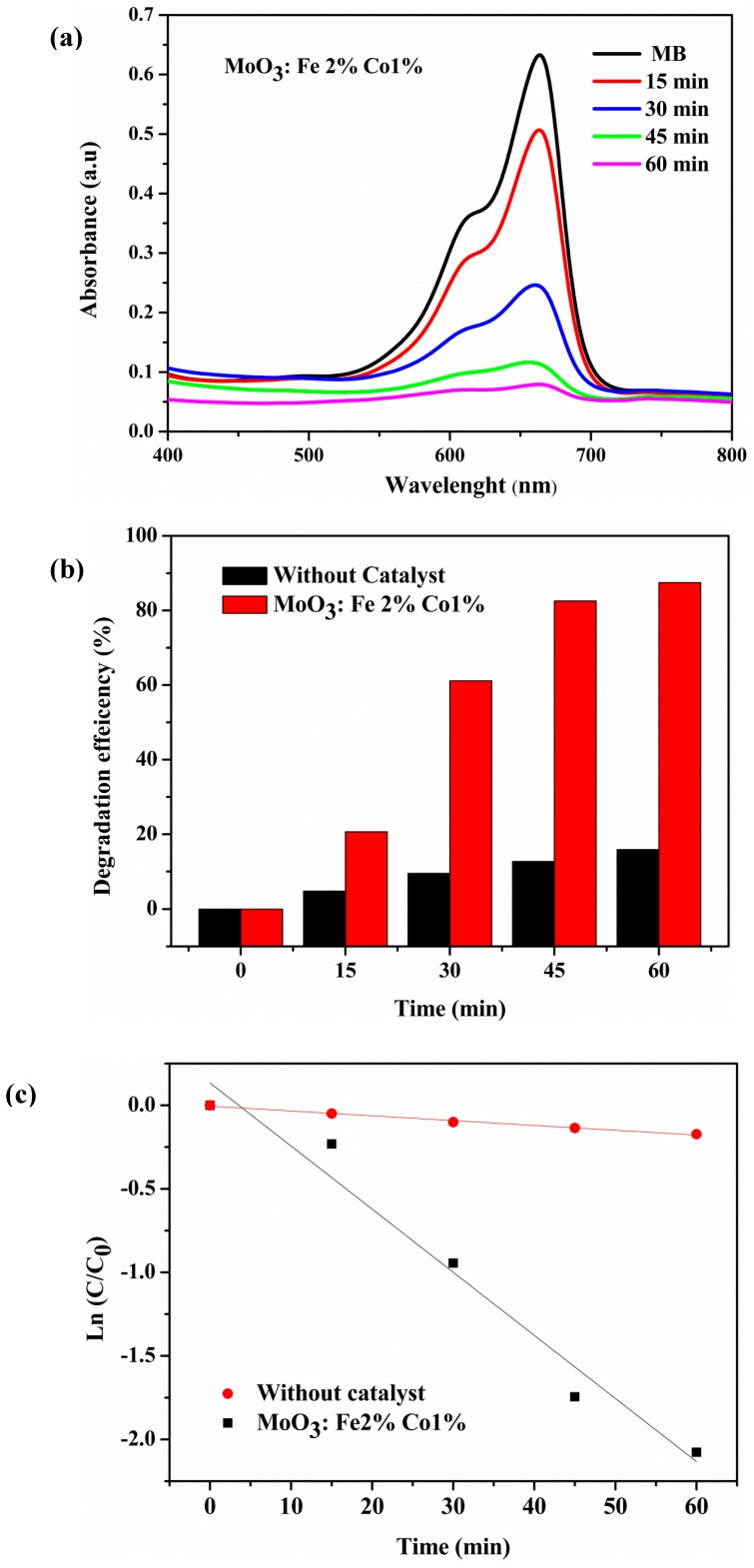
Time-dependent absorption spectra (**a**) of MB dye solution in the presence MoO_3_: Fe 2%-Co 1% (**b**) degradation efficiency and (**c**) degradation kinetics of MB dye.

In this work, the photocatalytic decomposition of methylene blue was used to test and compare the photocatalytic performance of the undoped and Fe-Co codoped MoO_3_. The absorption spectra of MB solution UV irradiated for 1 h is presented in Figure 11. There are two absorption peaks that correspond to methylene blue (MB), i.e., at 609 and 660 nm [53]. The peak located at 660 nm is so high for MoO_3_: Fe 2%-Co 2%, the intensity of this pic varies with Fe-Co content, but it becomes so low for MoO_3_: Fe 2%-Co 1% sample which indicates the best decomposition of MB dye solution [54]. Figure 12 presents the time-dependence of the absorption spectra (Figure 12a), the degradation efficiency (Figure 12b), and the degradation kinetics of MB solution in the presence MoO_3_: Fe 2%-Co 1% thin film (Figure 12c). After 1 h irradiation, about 90% of the MB amount was degraded by MoO_3_: Fe 2%-Co 1% (Figure 12a), compared to 35% degradation in the presence of the undoped MoO_3_ film.

The degradation efficiency of MoO_3_ (Fe,Co) thin films of MB dye is given in Figure 12b. The degradation efficiency of MoO_3_ (Fe,Co) was calculated using the following expression [55],

(12) $$\mathrm{Degradationefficiency}\left(\%\right)=\frac{C_{0}-C}{C_{0}}$$

where, *C*_0_ is the initial concentration of dye solution, and *C* is the concentration of dye solution after irradiation in the selected time interval [55].

The rate at which a pollutant is removed from the aqueous solution can be predicted by the reaction kinetics of MB dye, which can be was evaluated using Equation (13) [56]:

(13) $$Ln\frac{C}{C_{0}}=-kt$$

Figure 12c shows the plot of *Ln*(*C*/*C*_0_) versus the irradiation time. It can be observed that the *Ln*(*C*/*C*_0_) varies linearly with time, which indicates that the photodegradation of MB dye follows the first-order kinetics [56].

## 8. Conclusions

This comprehensive study on MoO_3_ and Fe-Co codoped MoO_3_ thin films obtained by spray pyrolysis presents results having a key information for industrial applications. The detailed nanostructural and morphological studies showed the formation of α-MoO_3_ structure with an interesting laminar crystallization within the layers, which forms nanoplates as observed by SEM, TEM, HRTEM, and SAED. While the Fe-Co codoping does not affect significantly the crystallite size, it increases the density of dislocation and stresses the film.

These crystallographic effects induced by doping are found in the optical studies. Transmission and reflection measurements have shown that the absorption edges of the film vary with doping concentration. The band gap energy value of the Fe-Co codoped MoO_3_ increases with Fe-Co codoping.

Electropyrolytic measurements performed on the thin films have demonstrated a metallic character that is not so sensitive to the Fe:Co ratio in the doped films. It is found that the thermal conductivity of MoO_3_ has a remarkable value of 24.1 Wm^−1^K^−1^ with a thermal diffusivity value of 3.8 × 10^−6^ m^2^s^−1^. Moreover, it is found that MoO_3_ codoped with 2% Fe-1% Co has the highest thermal conductivity. Due to their high thermal behavior, these results recommend the Fe-Co codoped molybdenum oxides as high-temperature thermoelectric materials.

MoO_3_ and Fe-Co Codoped MoO_3_ thin films present a rough surface with randomly oriented islet-like morphology, which is in particular important for the observed photocatalytic activity of MoO_3_: Fe 2%-Co 1%. The photodegradation measurements of the films against MB dye has shown a remarkable increase in the photosensitivity of MoO_3_: Fe 2%-Co 1%. We believe that the photosensitivity of the MoO_3_: Fe 2%-Co 1% thin film increase is due to a small excess in iron content, which could be explained by the following processes: (i) a small amount of iron could oxidize to produce Fe_2_O_3_ as a minority phase distributed randomly in the film, which improves the photocatalytic activity of the film; (ii) an excess of free carriers located near the conduction band could be formed in the MoO_3_: Fe 2%-Co 1% thin film, which enhances the photocatalysis behavior of the film; (iii) both phenomena could occur and the effect have cumulative enhancement action on the photocatalytic properties of the film.

This comprehensive study on undoped and Fe-Co codoped MoO_3_ thin films presents very interesting results especially those related to multifunctional properties such as optical, thermal and photocatalytic properties of this particular thin film system, which outweigh the increase in these proprieties, as would be expected from doping. Further studies are in progress to elucidate the remarkable effect of codoping on certain properties of MoO_3_ that are relevant to specific applications. Combined with low cost spray pyrolysis process, this technology has a considerable potential in reducing the cost of integrating metal oxide semiconductor thin films into devices.

## Figures and Tables

**Table 1:** Interplanar distance *d*_(*hkl*)_ of the MoO_3_: Fe-Co thin film with different codopant composition.

	*d*_(*hkl*)_ (Å)					
**(hkl)**	2*θ_hkl_*	MoO_3_	Fe 1% Co1%	Fe 1% Co 2%	Fe 2% Co 1%	Fe 2% Co 2%
**(020)**	12.73	6.95	6.94	6.94	6.94	6.94
**(040)**	25.58	3.48	3.47	3.47	3.47	3.47
**(131)**	27.29	3.26	3.26	3.26	3.26	3.26
**(261)**	38.88	2.31	2.31	2.31	2.31	2.31

**Table 2:** The texture coefficient *TC*(*hkl*) calculated for the Fe-Co doped MoO_3_ thin films.

	TC				
(hkl)	MoO_3_	MoO_3_: Fe 1% Co 1%	MoO_3_: Fe 1% Co 2%	MoO_3_: Fe 2% Co 1%	MoO_3_: Fe 2% Co 2%
**(020)**	1.78	1.63	1.64	1.68	1.51
**(040)**	1.40	1.38	1.46	1.46	1.55
**(131)**	0.30	0.46	0.36	0.28	0.32
**(261)**	0.52	0.53	0.54	0.58	0.62

**Table 3:** Caption of the stress (*ξ*), crystallite size (*D*) and dislocation density (*δ_dis_*) of MoO_3_: Fe-Co.

%Fe:%Co	*ξ* (10^−4^)	*D* (nm)	*δ_dis_* (10^13^ lines/m^2^)
**0**	44	82.7	15
**1:1**	48	77.4	17
**1:2**	50	71.1	20
**2:1**	55	65.1	24
**2:2**	46	69.2	21

**Table 4:** Characteristic energy lines for oxygen and molybdenum.

Element	O Kα	Mo Ll	Mo Lα1	Mo Lβ1	Mo Kα2	Mo Kα1	Mo Kβ1
Energy, keV	0.523	2.015	2.293	2.394	17.376	17.481	19.609

**Table 5:** Optical band gap energy, *E_g_*, and Urbach energy, *E_U_*, for different content of iron and cobalt in the spray solution.

%Fe:%Co	*E_g_* (eV)	*E_U_* (meV)
**MoO_3_**	3.75	370
**1:1**	3.80	218
**1:2**	3.85	245
**2:1**	3.88	200
**2:2**	3.95	150

**Table 6:** Thermal parameters of Fe-Co doped MoO_3_.

%Fe:%Co	Thermal Conductivity *K*, (W/mK)	Thermal Diffusivity *D*, (10^−6^ m/s)	Heat Capacity *C*, (10^6^ J/Km)	Thermal Effusivity *e* (10^3^ J/(Km^2^s^1/2^))
**0**	24.10 ± 0.02	3.80 ± 0.05	6.34	12.36
**1:1**	25.50 ± 0.03	4.10 ± 0.04	6.21	12.59
**1:2**	25.62 ± 0.03	4.65 ± 0.05	5.50	11.88
**2:1**	25.86 ± 0.04	5.15 ± 0.05	5.02	11.39
